# Widespread Expression of Hedgehog Pathway Components in a Large Panel of Human Tumor Cells and Inhibition of Tumor Growth by GANT61: Implications for Cancer Therapy

**DOI:** 10.3390/ijms19092682

**Published:** 2018-09-10

**Authors:** Jiri Réda, Jiri Vachtenheim, Kateřina Vlčková, Pavel Horák, Jiri Vachtenheim, Lubica Ondrušová

**Affiliations:** 1 Department of Transcription and Cell Signaling, Institute of Medical Biochemistry and Laboratory Diagnostics, Charles University Prague, 12108 Prague, Czech Republic; RedaJ@seznam.cz (J.R.); vlckova.katka@centrum.cz (K.V.); ppavel.hhorak@gmail.com (P.H.); lubica.ondrusova@gmail.com (L.O.); 2 Third Department of Surgery, First Faculty of Medicine, Charles University Prague and University Hospital Motol, 15006 Prague, Czech Republic; jcdv@seznam.cz

**Keywords:** Hedgehog, GLI, tumor cell lines, GANT61, apoptosis

## Abstract

The sonic Hedgehog/GLI signaling pathway (HH) is critical for maintaining tissue polarity in development and contributes to tumor stemness. Transcription factors GLI1–3 are the downstream effectors of HH and activate oncogenic targets. To explore the completeness of the expression of HH components in tumor cells, we performed a screen for all HH proteins in a wide spectrum of 56 tumor cell lines of various origin using Western blot analysis. Generally, all HH proteins were expressed. Important factors GLI1 and GLI2 were always expressed, only exceptionally one of them was lowered, suggesting the functionality of HH in all tumors tested. We determined the effect of a GLI inhibitor GANT61 on proliferation in 16 chosen cell lines. More than half of tumor cells were sensitive to GANT61 to various extents. GANT61 killed the sensitive cells through apoptosis. The inhibition of reporter activity containing 12xGLI consensus sites by GANT61 and cyclopamine roughly correlated with cell proliferation influenced by GANT61. Our results recognize the sensitivity of tumor cell types to GANT61 in cell culture and support a critical role for GLI factors in tumor progression through restraining apoptosis. The use of GANT61 in combined targeted therapy of sensitive tumors, such as melanomas, seems to be immensely helpful.

## 1. Introduction

The Hedgehog (HH) signaling pathway is a morphogenesis pathway crucial for the growth and patterning of various tissues during embryonic development [1,2]. The morphogen sonic Hedgehog binds the transmembrane receptor Patched (PTCH), which activates another transmembrane protein Smoothened (SMO) and triggers the HH pathway that influences the expression of many genes through the activation of transcription factors GLI1 and GLI2. GLI3 activates only exceptionally and behaves rather as a suppressor. HH components are highly conserved from fly to human [3]. Initially, the HH pathway was linked to the etiology of basal cell carcinoma and medulloblastoma [4,5,6,7,8]. The pathway transcriptionally upregulates the expression of survivin in more than half of analyzed cell lines [9]. Accumulating evidence suggests that the HH pathway is critical for almost all tumors. It has been found that HH signaling plays key roles in formation and maintenance of cancer stem cells (CSC), tumor stemness, and acquisition of epithelial-to-mesenchymal transition (EMT) in tumors. Since EMT is important and responsible for cancer cell invasion, metastasis, drug resistance, and tumor recurrence, the HH signaling pathway is now believed to be an important target for cancer therapy [10,11,12,13]. The HH pathway and GLI factors thus appear to be promising targets for cancer therapy [14]. Several cancers were shown to be sensitive to HH inhibition, such as lung cancer (both non-small cell lung cancer (NSCLC) [15,16,17,18] and small cell lung cancer (SCLC) [19,20]). Many reports highlight the importance of the HH pathway in pancreatic cancer and the usefulness of its inhibition [21,22,23,24]. The HH pathway was described to be crucial for the pancreatic cancer development and HH inhibition caused autophagy in CFPAC-1 cells in vivo and in mouse xenografts [25]. GLI1 promoted EMT and metastasis in pancreatic cells in a genome-wide screening study [26]. In many other cancer types, the HH pathway inhibition decreases the oncogenicity and has been beneficial for the patients. Melanomas critically require HH signaling [27,28,29], presumably with activated RAS-MAPK and AKT signaling cascades [27]. HH has been described to promote oncogenesis in leukemias [30,31,32,33,34], bladder cancer [35], and prostate cancer [36,37,38,39].

Global significance of the HH pathway for tumor initiation, progression, and metastasis is documented by additional literature. Mounting evidence indicates that HH signaling is required for the maintenance of glioblastoma and its CSC population [40,41]. GLI2 has been identified as a target for the treatment of osteosarcoma [42] and the HH pathway has been reported to be important for osteosarcoma progression and metastasis [43]. HH signaling produces self-renewal in embryonal rhabdomyosarcoma [44], has a critical role in the growth of neuroblastoma [45], ovarian cancer [46,47], hepatocellular carcinoma [48], colon carcinoma [49,50], and is pivotal for forming breast cancer CSC [51] and bone metastases [52]. Rhabdoid tumors and cell lines lack INI1 (SMARCB1/SNF5) tumor suppressor. This is a causative event in these tumors. This protein is central in the nucleosome remodeling complex SWI/SNF and is also rarely absent in rhabdomyosarcomas. It was found that INI1 binds GLI1. In the presence of INI1, the HH pathway is silent and the loss of INI1 triggers the activation of the HH pathway in rhabdoid tumors [53]. Ectopic INI1 is able to rescue the nonmalignant phenotype in rhabdoid tumor cell lines. This implies that an activated HH cascade causes this tumor type. This is intriguing because INI1 is present in all other cells including tumor cells with an elevated HH pathway activity (above). This implies a very specific cell context in rhabdoid tumors and suggests the HH pathway as a target for their treatment.

Several studies have implicated a noncanonical activation of the HH route in tumors, thus abrogating the necessity of upstream ligand signaling. Through this mechanism, GLI factors can be activated directly by many different mechanisms upregulated in tumor cells, predominantly operating in RAS/MAPK, Wnt, or AKT pathways [38,54,55,56,57]. As an example, KRAS activates GLI1 in pancreatic cancer cells [58], an androgen receptor (AR) protects GLI3 from proteolytic cleavage [38], and HH can be activated by the mTOR/S6K1 signaling [59]. This allows the processing of the deregulated HH pathway without the membrane signaling through direct aberrant GLI factors stimulation with the consequent expression of their prooncogenic targets. Here, we present results showing that the main components of the HH pathway are invariably expressed across a large panel of tumor cells of various cancer types. The most potent HH inhibitor GANT61 suppressed proliferation more or less in about half of tumor cell lines (the sensitive cells were eradicated presumably through apoptosis) and is a prime candidate as a compound for the combined therapy in many tumor types.

## 2. Results

### 2.1. Broad Expression of HH Cascade Components in Human Tumor Cell Lines

**Figure 1:**
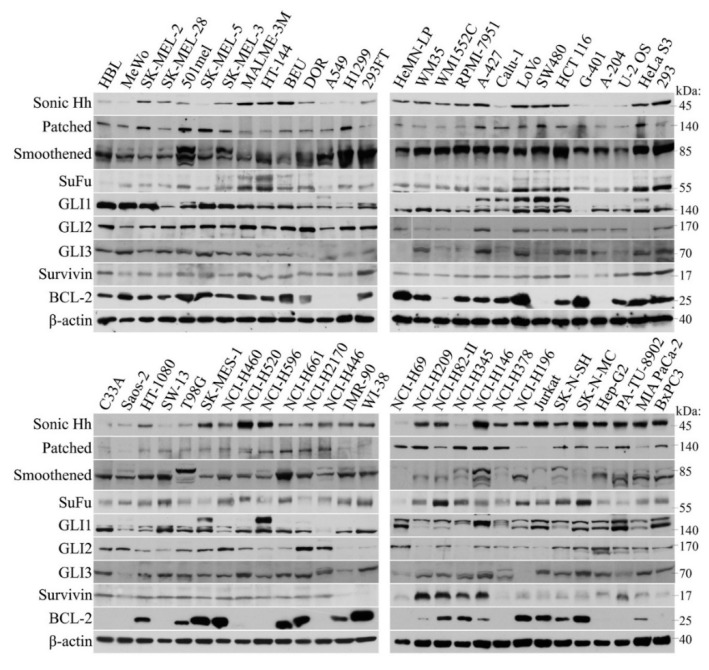
Panel of protein expression pattern of HH signaling components. Western blots made in RIPA extracts (30 μg) were probed with indicated antibodies. With some small exceptions, all HH proteins were expressed, although sometimes the expression level was weaker (see text). Survivin, an HH target, was invariably present in tumor cell lines. Notably, GLI3 was shown as a fragment that was cleaved off from the whole protein during sample preparation. However, its signals represent the true amount of intact GLI3 in the extract. The size of each protein in shown in kDa on the right.

We were interested in studying whether constituents of the HH pathway are invariably present in several tumor cell types or if some components are missing. It would potentially disable the activation of HH pathway in human cancer cell lines. A large screen has been performed and Western blots have shown complete expression of the main HH components in all tumor cells (Figure 1). Noteworthy, two lines expressed negligible GLI1 (G-401 and NCI H446), whereas GLI2 in them was expressed abundantly. In some other cells, GLI2 was low but GLI1 sufficiently expressed (RPMI-7951, Calu-1, HeLa S3, H-209, H-345, and Jurkat). The SuFu level was low in Hbl and H69 cells. In some tumor cell lines, expression of GLI3 was lower (DOR, Saos-2, and H-196). GLI3 is nevertheless only exceptionally necessary for processing of HH signals, whereas either GLI1 or GLI2 are generally required. Patched was weak in Saos-2 and Jurkat cells, and SMO was weakly expressed only in H-69 cells.

Very peculiar was a varying expression of the ligand sonic Hedgehog among the cell lines, irrespective of the tumor type. This nevertheless does not preclude the efficient functioning of the HH pathway, since, in view of the fact that HH is frequently activated noncanonically at the GLI factors level, the production of the ligand itself (acting by an autocrine or paracrine manner in cell lines) is dispensable. Three cell lines were nontransformed and tested for comparison with tumor cells (HeMN-LP, IMR-90, and WI-38). HeMN-LP (melanocytes) expressed both GLI2 and GLI1, but the two fibroblast cell lines expressed very low GLI2, but retained their GLI1 level. Expression of other components was retained in these normal cell lines. Survivin was present in all tumor cell lines. Our previous results have shown that in IMR90 cells, transfected GLI2 plasmid is capable of evoking the expression of endogenous survivin [9], which underlies the necessity of HH signaling for the survivin expression even in normal cells. BCL-2, another important antiapoptotic protein, was abundantly present in the majority of cell lines, however, in some tumors its expression was completely lacking, independently of the tumor type. Together, the widespread abundance of HH components indirectly support the importance of the HH signaling in tumors and is in accord with the previous results.

### 2.2. Inhibition of Cell Proliferation by GLI Inhibitor GANT61

**Figure 2:**
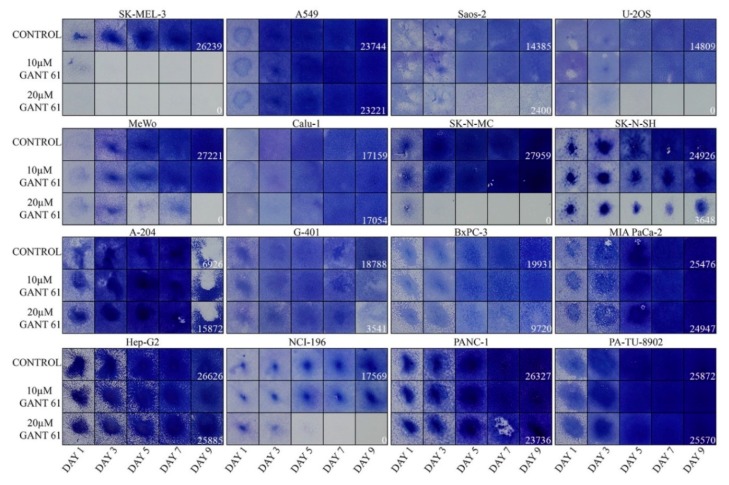
Proliferation assays showing the sensitivity to GANT61. The intensity of staining with crystal violet indicates the relative number of cells. The quantification numbers are given only for day 9 for controls and GANT61 (20 μM) as these fields were the most important outcome of the experiment. Please note that the lower number of A-204 control cells at day 9 is caused by cell detachment. Two experiments with similar results were performed and one is presented. Results are shown as squares cut from the 12-well plate wells.

We next tested the sensitivity to a GLI inhibitor GANT61 in a panel of 16 tumor cell lines (Figure 2). The tumor types included melanomas, NSCLC and SCLC, osteosarsomas, neuroblastomas, rhabdoid tumors, hepatocellular carcinoma, and pancreatic cancers. Some cells were eradicated completely at the end of the experiment (SK-MEL-3, U-2 OS, MeWo, SK-N-MC, and H196). Another group of cells was only partially sensitive to GANT61 under the experimental conditions (Saos-2, SK-N-SH, G-401, and BxPC-3). The remaining cell lines did not reveal any sensitivity when cultured in GANT61 (A549, Calu-1, A-201, Hep-G2, and the three pancreatic cancer cell lines MIA PaCa-2, PANC-1, and PA-TU-8902). The pancreatic tumors were surprisingly most resistant to GANT61 treatment, although previous reports describe their sensitivity to the blocking of HH signaling [21,60].

Expectedly, melanomas were sensitive to GANT61 (Figure 2). We have previously tested melanoma cells and found that GANT61 was variably effective in all tumors. The combination with obatoclax (a BCL-2 family inhibitor) revealed a better effect, showing clear synthetic lethality in six of nine melanoma lines [29] (Figure S1). The most sensitive cell line was SK-MEL-3. Here, less responsive were two osteosarcomas and one SCLC. Also, G-401 was sensitive, but only at day 9. Two neuroblastoma cell lines responded to GANT61 as well. Other cell lines did not reveal any GANT61 sensitivity even after day 9 (A-204, Hep-G2, NSCLC, and pancreatic cell lines from which only BxPC-3 reacted slightly, Figure 2). It is important to note that with the exception of the extremely sensitive SK-MEL-3, all other cells responded only to 20 μM GANT61 and were insensitive to a 10 μM concentration. We can speculate that higher doses of GANT61 or a prolonged time of treatment would have a better effect in eradicating tumor cells. In our assays, longer incubation time was precluded as untreated control cells would overgrow and detach. Our findings suggest that the testing of cancer cell types might be useful for further consideration of therapy and show that more than half of tested tumors (when we include melanoma cells from Figure S1) were more or less sensitive to 20 μM of GANT61 when observed up to 9 days.

### 2.3. GANT61 Eradicates Tumor Cells through Apoptosis

**Figure 3:**
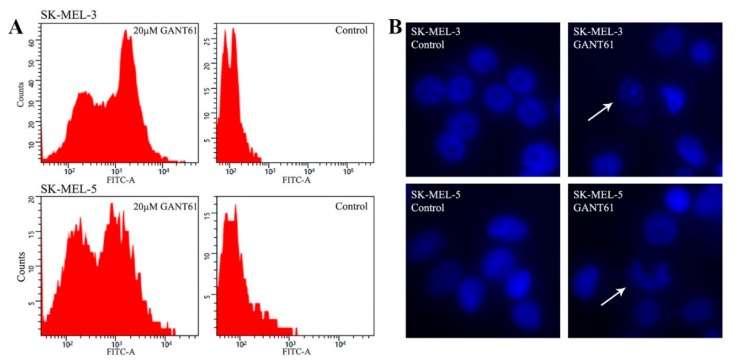
(**A**) TUNEL assay detecting apoptosis in two cell lines. Cells were seeded on 60-mm dishes, and the next day, 20 μM GANT61 was added. The normal medium was replaced in controls. After three days, the majority of cells treated with GANT61 detached in both SK-MEL-3 and SK-MEL-5 cells. Both detached and remaining attached cells were used for analysis. FITC fluorescence clearly shows massive apoptosis in GANT61-treated cells. The percentage of the apoptotic and nonapoptotic cells were calculated using ImageJ software (National Institutes of Health, Bethesda, MD, USA). The results of cell quantification were as follows. SK-MEL-3 cells treated with GANT61: apoptotic cells 62.62%, nonapoptotic cells 37.38%; SK-MEL-3 controls: apoptotic cells 0.4%, nonapoptotic cells 99.6%. SK-MEL-5 cells treated with GANT61: apoptotic cells 51.97%, nonapoptotic cells 48.03%; SK-MEL-5 controls: apoptotic cells 4.18%, nonapoptotic cells 95.82%. No cell cycle blockade was observed. (**B**) Fluorescence showing apoptotic nuclei in the same cells as in (**A**), treated equally with GANT61 or untreated (control cells). Cells were mounted in a medium containing DAPI and documented by fluorescence. Magnification: 200×. Arrows show apoptotic nuclei.

To gain insight into the mechanism underlying the eradication of cells in proliferation assays, we carried out the TUNEL assay that detects apoptosis. Many previous papers indicate that GANT61 kills the cells through apoptosis [29,49,57,61]. We have chosen two GANT61-sensitive tumors cell lines, SK-MEL-3 (see Figure 2) and SK-MEL-5 (see Figure S1). Cells were treated with 20 μM GANT61 for 3 days and both detached and attached cells were combined and analyzed using flow cytometry. The extent of apoptosis was analyzed by a TUNEL assay (Figure 3A). The GANT61-treated cells revealed massive apoptosis (reflected by the FITC staining, about 60% of apoptotic cells in SK-MEL-3 and 50% in SK-MEL-5 cells, right peaks, left panels, Figure 3A), while negligible apoptosis was observed in control cells. No cell cycle alteration was seen. We thus presume that no stable blockade of the cell cycle occurred, as the cells stepwise disappeared, although sometimes slowly, which was caused by cell detachment. Since it has been reported that GANT61 may cause autophagy in some cells types [25,62], it can also be possible that in some cell lines, the elimination of cells could be brought about by autophagy. However, it is highly probable that most cells were eradicated by apoptosis as it is a well-known consequence of GANT61 treatment. To corroborate the results in Figure 3A, we left the same cells in a normal medium or medium with 20 μM GANT61 for 3 days, fixed the cells, and mounted in DAPI-containing medium. Apoptotic figures were seen in both cell types, whereas no apoptotic nuclei were present in controls (Figure 3B).

### 2.4. Activity of the Promoter Containing 12xGLI Consensus Site

**Figure 4:**
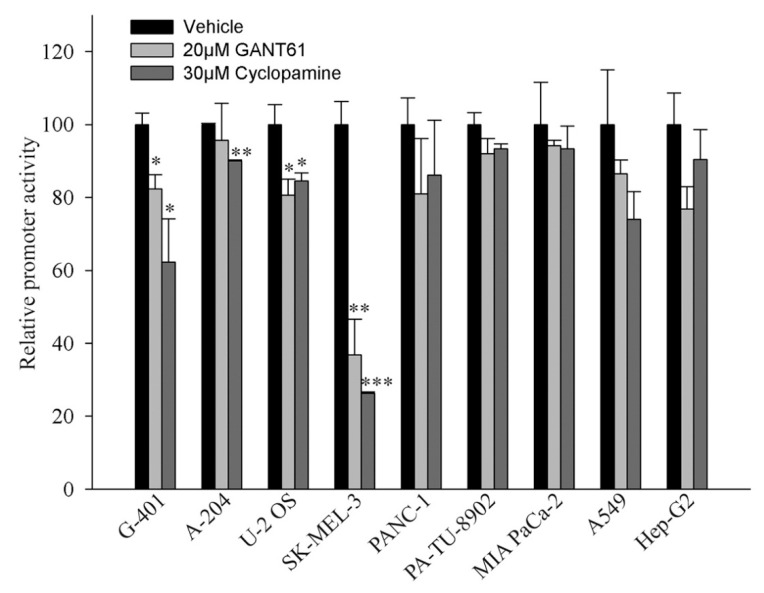
GANT61 and cyclopamine slightly reduced the 12xGLI reporter activity. Cells were seeded in 12-well plates and transfected the next day with the 12xGLI-luciferase plasmid together with a *Renilla luciferase* plasmid for the correction of transfection efficiency. The next day, inhibitors were added to the indicated concentration and cells were harvested 20 h later. No cell deterioration was observed after this period, even in sensitive SK-MEL-3 cells. The experiment was performed twice in triplicates with similar results and one experiment is presented. Data are presented as mean + SD. No mark means insignificant, statistical significance is: * *p* < 0.05, ** *p* < 0.01, *** *p* < 0.001.

To study whether a GLI-responsive promoter-reporter is also affected by HH inhibitors GANT61 and cyclopamine, 12xGLI-luciferase reporter and a reference plasmid were cotransfected in several cell lines that were either variably responsive or nonresponsive to GANT61 in proliferation assays. As shown in Figure 4, the sensitive SK-MEL-3 cells were inhibited by cylopamine and GANT61 extensively. To a lesser extent, reporter activity in G-401, A-204, and U-2 OS was also inhibited. Of these cells, U-2 OS were eradicated from day 5 onwards in the proliferation assay, G-401 were diminished only on day 9, and A-204 were resistant in the proliferation assay (Figure 2). The inhibition of the reporter by GANT61 or cyclopamine was insignificant in other cell lines (PANC-1, PA-TU-8902, MIA-PaCa-2, A-549, and Hep-G2). These cells were also completely resistant in the proliferation assay (Figure 2). The reporter activity thus approximately mimicked the sensitivity of cells to GANT61 (A-204 cells were only negligibly, though significantly, inhibited by cyclopamine, due to very low +SD, and were resistant to GANT61 in proliferation assay). Together, the results indicate a correlation between the sensitivity to inhibitors in the reporter assay and the sensitivity to GANT61 in longer proliferation analysis.

## 3. Discussion

The HH signaling pathway, acting through transcription factors GLI1, GLI2, and GLI3, has been identified as critical for the initiation and progression of a number of cancers. Originally, it was believed to be important for only basal cell carcinoma (BCC) and meduloblastoma. Gradually, the pathway becomes a crucial signaling pathway for all frequent cancer types with the GLI family transcription factors being essential in tumor initiation, progression, EMT, CSC, and metastasis, dependent on the tumor cell context. HH signaling is a network rather than as a simple linear pathway because of its cooperation with many other cell signaling pathways and its frequent noncanonical activation. GLI factors have several oncogenic targets [63]. Recently, using a large tumor panel, we identified survivin as another important GLI2 target in more than half of tumor cell types [9], suggesting a synergy in HH and survivin in forming tumors stemness and maintaining CSC. This implies more effective therapy by combining HH and survivin inhibitors.

Here, we have first analyzed the expression of HH cascade components across a panel of 56 tumor types using Western blot analysis. It was found that they are generally expressed (only exceptionally showing lower expression level). Importantly, either GLI1 or GLI2 were always present in all samples. In three normal control cell lines, the HH proteins were also present. HH signaling is emerging to be essential for the progression of nearly all tumors [12,13]. The presence of its components is therefore required for the proper progression of the pathway. In proliferation assays, GANT61 was active in melanoma cells (Figure 2 and Figure S1) and also in several other tumor cell lines. The most resistant seemed to be NSCLC and pancreatic cancer cells. This was rather surprising as many reports describe the blockage of the HH pathway in the treatment of pancreatic cancer in preclinical and clinical settings. In tumors, the dense impenetrable stroma is mixed with the pancreatic cancer cells in vivo, due to which, drugs cannot invade across this physical barrier, and that may cause a drug resistance [22,64,65,66]. Since in cell lines the stroma is missing, the drugs should have better access to tumor cells and the druggability might be more feasible. As GANT61 appeared to be nonfunctional in eradicating pancreatic tumor cells, the HH pathway possibly needs, e.g., a second agent to achieve cell killing. A possible explanation could also be that the cell lines used here have not been sensitive to GANT61, while other cell lines (not tested) might have been responsive. In pancreatic tumors, the situation might be even more complicated, e.g., because stromal cells themselves produce Hedgehog and HGF that support the tumor growth [67]. It requires further clarification why in pancreatic cancer the HH pathway sensitivity to drugs in vivo has specific requirements in which tumor stroma is determining, causing the known resilience and drug resistance of these tumors.

Our results suggest which type of cancer is resistant or sensitive to GANT61 when it is applied directly on cells in culture (Figure 2). Malignant melanomas are sensitive, when taken into account also our previous results (Figure S1). Thus, GLI factors are important to contribute to keeping their antiapoptotic status. It is believed that MITF (microphthalmia-associated trancription factor), a key factor in melanoma transcription circuitry, maintains antiapoptosis in melanomas [68]. It has been nevertheless demonstrated that low-MITF melanoma cell lines can also proliferate very fast, implicating sufficient antiapoptotic protection [29,69]. HH-GLI signaling has been recognized to keep melanoma stemness and maintain the presence of CSC [70]. Furthermore, the two neuroblastoma cell lines and one SCLC cell line were also relatively sensitive to GANT61, whereas two NSCLC were resistant. In GANT61-resistant cells, antiapoptotic signals ensuring tumor progression can maintain apoptosis by other pathways. Reporter assays measuring the sensitivity of the 12xGLI consensus promoter to GANT61 and cyclopamine roughly correlated with cell proliferation. Our results suggest that HH signaling participates in preventing cell death perhaps in more than half of all tumors cell lines. We speculate that the situation might be similar in other tumor cell lines as well. Taken together, HH signaling plays an important role in preventing tumors cell apoptosis in some cancer cell types.

## 4. Materials and Methods

### 4.1. Cell Cultivation

Cells were maintained in appropriate media (DMEM or RPMI1640) supplemented with 10% fetal calf serum (Gibco, Waltham, MA, USA), l-glutamine, streptomycin, and penicillin (Sigma, St. Louis, MO, USA). Some cells were cultured in EMEM medium supplemented also with essential amino acids and pyruvate (Sigma). Fresh media were replaced every third day. HH inhibitors GANT61 or cyclopamine were present in media as indicated in Figures and Figure legends. All melanoma cell lines were maintained in RPMI1640 medium with the exception of lines WM35 and WM1552C that were kept in DMEM. NSCLC and SCLC cell lines cells were grown in RPMI1640 medium with the exception of Calu-1 (DMEM). SK-N-SH, SH-N-MC, HT-1080, and T98G cells were maintained in EMEM. The remaining cell lines were grown in DMEM medium.

### 4.2. Cell Lines

All cell lines were of human origin. Melanoma cell lines DOR, Beu, and Hbl were previously described [29]. Other melanoma cell lines (MeWo, SK-MEL-2, SK-MEL-28, SK-MEL-5, SK-MEL-3, Malme 3M, HT144, WM35, WM1552C, and RPMI-7931) were purchased from American Type Culture Collection (ATCC) (Manassas, VA, USA). Normal human melanocytes HeMN-LP were from Cascade Biologics (Portland, OR, USA). NSCLC lung cancer cell lines A549, HT1299, A-427, Calu-1, H-460, H-520, H596, H-661, H-2170, and SK-MES-1, and SCLC cell lines H-446, H-69, H-209, H-82, H-345, H-146, H-378, H-196 were purchased from ATCC. 293FT cells were from Invitrogen (Carlsbad, CA, USA). Colorectal cell lines LoVo, SW480, HCT116 were from ATCC. All other cell lines were purchased also from ATCC: G-401 and A-204 (rhabdoid tumors), U-2 OS and Saos-2 (osteosarcomas), HeLa S3 and C33A (cervical carcinomas), 293 (renal carcinoma), HT-1080 (connective tissue fibrosarcoma), SW-13 (adrenal gland carcinoma), T98G (glioblastoma), IMR90 and WI-38 (normal human fibroblasts), Jurkat (T-cell leukemia), Hep-G2 (hepatocellular carcinoma), SK-N-SH and SH-N-MC (neuroblastomas), PANC-1, PA-TU-8902, MIA PaCa-2, and BxPC-3 (pancreatic carcinomas).

### 4.3. Western Blots

Commercially available primary antibodies used were as follows: Sonic Hedgehog, Cell Signaling Technology #2207 (Danvers, MA, USA); Patched, Biorbyt #157169 (San Francisco, CA, USA); SMO, #ab72130 (Abcam, Cambridge, UK); SuFu, Cell Signaling #2520; Gli, Abcam #ab134906; Gli2, #sc-271786 (Santa Cruz Biotechnology, Dallas, TX, USA); Gli3, Biorbyt #157158; Survivin, Santa Cruz #sc-17779; BCL-2, BD Pharmingen #556354 (San Jose, CA, USA); β-actin, Sigma #A5316. HRP-labelled second antibodies were from Cell Signaling.

For Western blot analysis, cells were lysed in RIPA buffer (50 mM Tris-HCl pH 7.5, 150 mM NaCl, 5 mM EDTA, 1% NP-40, 0.5% sodium deoxycholate, 0.1% SDS), supplemented with aprotinin, leupeptin, pepstatin (Sigma), COMPLETE, and PhoStop (Roche, IN, USA). Total lysates containing 30 μg of protein were separated on SDS-PAGE gels and subsequently transferred onto a PVDF membrane (Millipore, Billerica, MA, USA). Membranes were then subjected to probing with antibodies. Western blot signals were detected by using SuperSignal West Pico Chemiluminescent substrate (Fisher Scientific, Waltham, MA, USA) and exposed on films.

### 4.4. Proliferation Assays

To perform proliferation assays, colony outgrowth assays were carried out. Cells were trypsinized and seeded in about 40–50% confluency on 12-well plates (day 0). The next day (day 1), cell lines were treated with 10 μM GANT61 or 20 μM GANT61 (SelleckChem, München, Germany), for a maximum of 9 days. The medium was refreshed every third day. The plates were then fixed in 3% paraformaldehyde solution in 1× PBS and stained with 1% crystal violet and photodocumented. Two most important fields (day 9, control and 20 μM GANT61) were quantitated using ImageJ software. Two experiments were performed in duplicate. Results of both experiments were similar.

### 4.5. Detection of Apoptosis

A TUNEL (terminal deoxynucleotidyltransferase-mediated dUTP nick-end labelling) assay was performed according to the manufacturer’s instructions (BD Biosciences, San Jose, CA, USA). Results of FITC staining were analyzed on a flow cytometer BriCyte EA (Mindray, Shenzhen, China). A total of 50,000 cells were analyzed in each sample. The number of apoptotic cells was determined using ImageJ software. 

### 4.6. Microscopic Detection of Apoptotic Nuclei

Immunofluorescence assays were performed as described previously [71]. Briefly, cells were seeded in NUNC (Roskilde, Denmark) chambers, 20 μM GANT61 added next day and treated (or untreated, controls) for three days, and mounted in a DAPI-containing medium. Images of nuclear apoptotis figures and controls were there taken using a fluorescent microscope.

### 4.7. Reporter Assays

Luciferase Reporter Gene Assay: Luciferase reporter plasmid with luciferase gene under the transcriptional control of 12xGLI full consensus was obtained from Prof. R. Toftgard (Karolinska Institutet, Stockholm, Sweden). After transfection of the plasmid (1 μg), together with the *Renilla luciferase* reporter plasmid (as a reference for transfection efficiency) on the 12-well plates in triplicates, the inhibitors GANT61 and cyclopamine were added at concentrations indicated in Figure 4 for 20 h. Cells were then harvested and the reporter activity was measured using a dual luciferase kit (Promega, Madison, WI, USA) according to the instructions of the manufacturer. Statistical significance is shown in the Figure 4. Two experiments were performed and one is presented. Results of both experiments were similar.

### 4.8. Statistical Analysis

To calculate the statistical significance of the reporter assays, a two-tailed Student test was used. The *p* values are listed in the corresponding figure legend. In all figures the error bars represent mean + SE. Proliferation assays and TUNEL assay were quantified by ImageJ software (National Institutes of Health, Bethesda, MD, USA).

## Supplementary Materials

The following are available online at http://www.mdpi.com/1422-0067/19/9/2682/s1.

## Abbreviations

CSC	cancer stem cells
HRP	horseradish peroxidase
DAPI	40,6-Diamidino-2-Phenylindole, Dihydrochloride
SMARCB1	SWI/SNF related, matrix associated, actin dependent regulator of chromatin, subfamily b, member 1
EMT	epithelial-to-mesenchymal transition
TUNEL	terminal deoxynucleotidyl transferase-mediated d-UTP Nick End Labeling
AR	androgen receptor
BCC	basal cell carcinoma
GLI	glioma family zinc finger protein
MITF	microphthalmia-associated trancription factor
HGF	hepatocyte growth factor
PTCH	patched
SMO	smoothened, frizzled class receptor
NSCLC	Non-small cell lung cancer
SCLC	Small cell lung cancer
SWI/SNF	SWItch/Sucrose Non-Fermentable
mTOR	mechanistic target of rapamycin
FITC	fluorescein isothiocyanate
