# Supplementary material for: Widespread Expression of Hedgehog Pathway Components in a Large Panel of Human Tumor Cells and Inhibition of Tumor Growth by GANT61: Implications for Cancer Therapy
Source: Int J Mol Sci. 2018 Sep 10;19(9):2682. doi: 10.3390/ijms19092682 (PMC6163708; doi:10.3390/ijms19092682)
Supplement: Supplementary file 1 [file ijms-19-02682-s001.pdf]

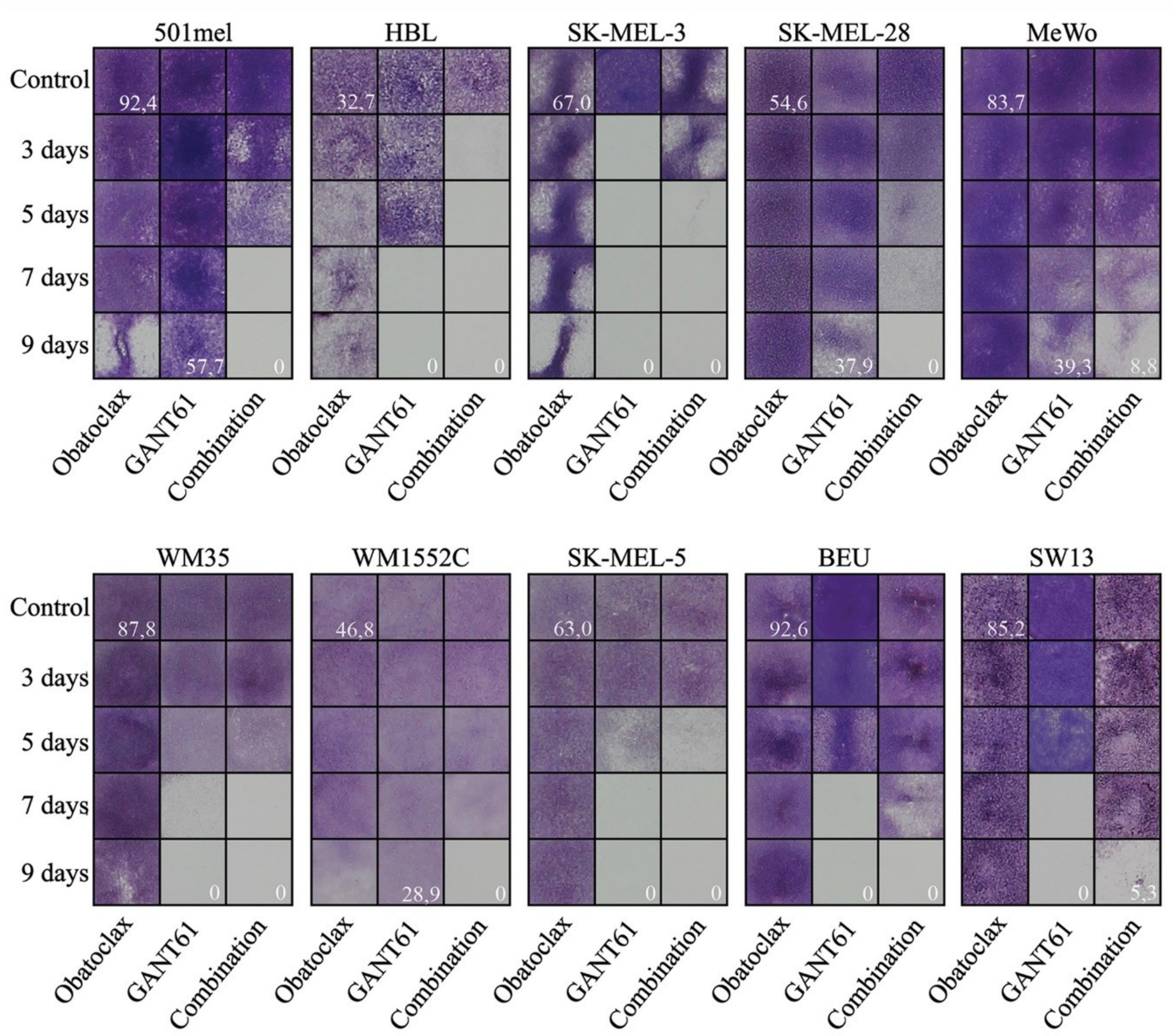

Figure S1. Synthetic lethality of most melanoma cell lines in a combined treatment. Sensitivity of melanoma cells to 20μM GANT61, 300 nM obatoclax, or their combination is shown. The picture is a Figure 4 from the published paper [29] shown with a kind permission of the publisher (Spandidos Publications).
